# Therapeutic efficacy of generic artemether–lumefantrine in the treatment of uncomplicated malaria in Ghana: assessing anti-malarial efficacy amidst pharmacogenetic variations

**DOI:** 10.1186/s12936-024-04930-1

**Published:** 2024-04-29

**Authors:** Nicholas Ekow Thomford, Tracy Kellermann, Robert Peter Biney, Charné Dixon, Samuel Badu Nyarko, Richmond Owusu Ateko, Martins Ekor, George B. Kyei

**Affiliations:** 1 Pharmacogenomics and Genomic Medicine Group, Department of Medical Biochemistry, School of Medical Sciences, College of Health and Allied Sciences, University of Cape Coast, Cape Coast, Ghana; 2 Division of Human Genetics, Department of Pathology, Faculty of Health Sciences, University of Cape Town, Anzio Road, Observatory, Cape Town, 7925 South Africa; 3 Division of Clinical Pharmacology, Faculty of Medicine and Health Sciences, Stellenbosch University, Cape Town, South Africa; 4 Department of Pharmacotherpaeutics and Pharmacy Practice, School of Pharmacy and Pharmaceutical Sciences, University of Cape Coast, Cape Coast, Ghana; 5 Department of Chemical Pathology, University of Ghana Medical School, University of Ghana, Legon, Accra, Ghana; 6 Division of Chemical Pathology, Department of Pathology, Faculty of Health Sciences, University of Cape Town, Cape Town, South Africa; 7 Department of Pharmacology, School of Medical Sciences, College of Health and Allied Sciences, University of Cape Coast, Cape Coast, Ghana; 8 Department of Virology, Noguchi Memorial Institute for Medical Research, University of Ghana, Legon, Ghana; 9 Department of Medicine, Washington University School of Medicine, St. Louis, MO 63110 USA

**Keywords:** Pharmacogenomics, Pharmacokinetics, Generic anti-malarials, Artemether–lumefantrine, CYP2B6, CYP3A5

## Abstract

**Background:**

Despite efforts made to reduce morbidity and mortality associated with malaria, especially in sub-Saharan Africa, malaria continues to be a public health concern that requires innovative efforts to reach the WHO-set zero malaria agenda. Among the innovations is the use of artemisinin-based combination therapy (ACT) that is effective against *Plasmodium falciparum*. Generic artemether–lumefantrine (AL) is used to treat uncomplicated malaria after appropriate diagnosis. AL is metabolized by the cytochrome P450 family of enzymes, such as CYP2B6, CYP3A4 and CYP3A5, which can be under pharmacogenetic influence. Pharmacogenetics affecting AL metabolism, significantly influence the overall anti-malarial activity leading to variable therapeutic efficacy. This study focused on generic AL drugs used in malarial treatment as prescribed at health facilities and evaluated pharmacogenomic influences on their efficacy.

**Methods:**

Patients who have been diagnosed with malaria and confirmed through RDT and microscopy were recruited in this study. Blood samples were taken on days 1, 2, 3 and 7 for parasite count and blood levels of lumefantrine, artemisinin, desbutyl-lumefantrine (DBL), and dihydroartemisinin (DHA), the active metabolites of lumefantrine and artemether, respectively, were analysed using established methods. Pharmacogene variation analysis was undertaken using iPLEX microarray and PCR–RFLP.

**Results:**

A total of 52 patients completed the study. Median parasite density from day 1 to 7 ranged from 0–2666/μL of blood, with days 3 and 7 recording 0 parasite density. Highest median plasma concentration for lumefantrine and desbutyl lumefantrine, which are the long-acting components of artemisinin-based combinations, was 4123.75 ng/mL and 35.87 ng/mL, respectively. Day 7 plasma lumefantrine concentration across all generic ACT brands was ≥ 200 ng/mL which potentially accounted for the parasitaemia profile observed. Monomorphism was observed for *CYP3A4* variants, while there were observed variations in *CYP2B6* and *CYP3A5* alleles. Among the *CYP3A5* genotypes, significant differences in genotypes and plasma concentration for DBL were seen on day 3 between 1/*1 versus *1/*6 (p = 0.002), *1/*3 versus *1/*6 (p = 0.006) and *1/*7 versus *1/*6 (p = 0.008). Day 7 plasma DBL concentrations showed a significant difference between *1/*6 and *1/*3 (*p* = *0.026*) expressors.

**Conclusions:**

The study findings show that *CYP2B6* and *CYP3A5* pharmacogenetic variations may lead to higher plasma exposure of AL metabolites.

## Background

Despite the efforts and significant investment in malaria eradication in sub-Saharan Africa (SSA), the disease remains a major public health challenge [1, 2]. Africa, in general, carries a more significant proportion of the global malaria burden accounting for 95% of malaria cases and 96% of malaria-related mortality [3]. Recent publications and the reports on malaria of the World Health Organization (WHO) have documented a decline in malaria morbidity and mortality [4–7], although it continues to remain a public health challenge.

Malaria is endemic and perennial in Ghana, with a pronounced seasonal variation. Malaria is highly prevalent during the rainy season, providing the perfect environment for the female *Anopheles* mosquito. Ghana is one of the eleven countries that accounted for 70% of the global malaria cases and 71% of estimated deaths in 2017 [4]. Across Ghana, the incidence per 1000 people decreased by 7.8% each year from 2011–2018. However, there was a decline in incidence/1000 people to 2.6% from 2018–2020 [8].

Accurate diagnosis and timely treatment with effective anti-malarial medication are major tools in malaria control. Over the years, several anti-malarial medications have been rolled out and changed over time due to the emergence of *Plasmodium falciparum* resistance and undesirable side effects. Anti-malarial medications from quinine to chloroquine, mefloquine, sulfadoxine–pyrimethamine (SP) and currently artemisinin-based combination therapy (ACT) have been prescribed for malaria treatment over decades. Ghana implemented its ACT policy in 2004 with the rollout of artesunate–amodiaquine as the first-line drug to replace chloroquine which was no longer effective due to extensive *P*. *falciparum* resistance across the country and Africa [9]. In 2009, there was a change in the anti-malarial drug policy of ACT to include artemether–lumefantrine and dihydroartemisinin–piperaquine as alternative first-line treatment medications due to challenges with tolerability to artesunate–amodiaquine by a significant section of the Ghanaian population [10].

Some gains have been made since the roll out of the anti-malarial drug policy of ACT. However, the continued success of the ACT policy and its subsequent implementation will largely depend on the availability, quality and cost of ACT drugs and the ability of health professionals to adhere to treatment guidelines [11–13]. The Affordable Medicine Facility for Malaria Initiative (AMFm) assisted in expanding access to ACT and promoting the appropriate use of antimalarial medication [14, 15]. From the AMFm’s initiatives, which was largely seen as success, Coartem® (Novartis Pharma AG, Basel Switzerland) became an innovator product with significant anti-malarial activity used for malaria treatment [16]. However, the high cost and availability of Coartem® has called for alternative, equally efficacious but affordable, anti-malarial medications that can hopefully elicit enough anti-plasmodial activity. The Food and Drug Administration of Ghana has approved several generic artemether–lumefantrine (AL) anti-malarial medications for use in Ghana, including Lumether (20 mg/120 mg), Luzatil (20 mg/120 mg), Artetab (80 mg/480 mg), Lumetrust (80 mg/480 mg) and Shal’Artem (20 mg/120 mg). These generic drugs are prescribed at health facilities and pharmacies for malaria treatment after rapid detection tests and or microscopy. AL is prescribed in individuals of weight ≥ 35 kg or ≥ 12 years of age as an 8–12 hourly, 3-day dosage regimen of 80 mg/480 mg artemether/lumefantrine. The number of AL tablets are adjusted to the required 80 mg/480 mg for ALs of 20 mg/120 mg (4 tablets) and ALs of 40 mg/240 mg (2 tablets) depending on the brand.

Artemether has a fast absorption rate with a rapid plasma clearance (T_1/2_ = 2–3 h) with it major metabolite dihydroartemisinin (DHA) following a similar pattern. Lumefantrine is slowly absorbed followed by a slow plasma clearance rate (T_1/2_ = upto 10 days). Intake of fatty food increases bioavailability of AL by approximately 2–tenfold [17, 18]. AL is metabolized by the CYP450 enzymes such as CYP2B6, CYP3A4/5, CYP2A6 and UGTs to active components to exert their therapeutic effects. AL metabolism leads to active metabolites of desbutyl-lumefantrine (DBL) and dihydroartemisinin (DHA) which provides its therapeutic anti-malarial activity [19]. Most of the presently available evidence point to pharmacogenetic variations in these drug metabolizing enzymes, especially cytochrome P450, and have been shown to influence drug disposition and efficacy [20]. The importance of pharmacogenetics in AL is clearly seen in circumstances of resistance, shrinking narrow therapeutic window and efficacy [21]. Generic AL is critical in the management and control of malaria in low- and middle-income countries (LMICs) such as Ghana as they are affordable and readily available. However, substandard generic drugs expose parasites to sub-therapeutic drug pressure, which enables *P. falciparum* resistance selection and treatment failures and threatens patients’ safety [22, 23].

A recent study in Tanzania has explored the activity of generic drugs used in treating uncomplicated malaria in comparison to the innovator drug Coartem® [24]. Therapeutic efficacy of drugs, including artemisinin-based combinations, depends on specified plasma concentrations of active drugs or metabolites [25, 26]. Plasma concentration of drugs and metabolites depends on several pharmacokinetic considerations, including variations on genetic profiles of individuals. There is very little data on plasma metabolite profiles in most medications administered among the Ghanaian population. Bearing in mind the significant role of genetics on plasma concentration of reports on malaria and the economics of malaria management in the country *vis-a-vis* sub-optimal therapeutic outcomes in some generic anti-malarials, our study focused on potential pharmacogenetic variations of response to generic drugs used in anti-malarial treatment as prescribed at health facilities and by evaluating the pharmacogenomic influences on their efficacy.

## Methods

### Study design, site and participants

**Fig. 1:**
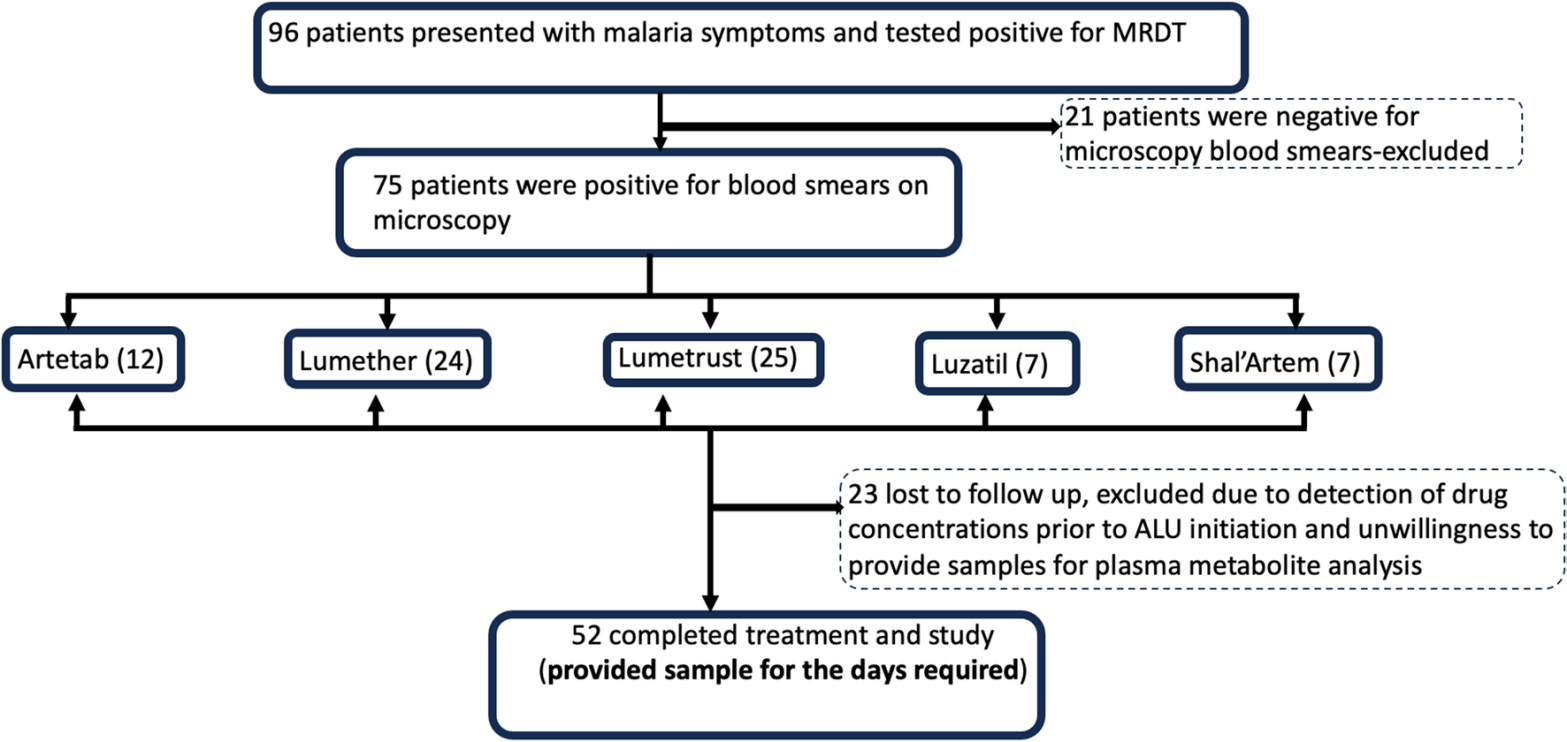
Study recruitment, follow-up, and sampling procedure

This was prospective open-label pharmacogenomic-pharmacokinetic study conducted to compare the metabolite profiles and treatment outcomes in patients being treated with generic artemether–lumefantrine medications for the treatment of uncomplicated P. *falciparum* malaria. Patients were selected after diagnosis for uncomplicated malaria (defined as “a patient who presents with symptoms of malaria and a positive parasitological test (RDT and or microscopy), but with no clinical features of severe malaria”) [27], age 15 years and above and for females not pregnant. Patients were excluded if they had severe malaria [28], other significant health conditions, were taking medications that could potentially interact with the AL (e.g. rifampin, carbamazepine, phenytoin, St. John’s work) or had initiated treatment of malaria with either herbal or other ACTs prior to the recruitment. Of the 96 patients who presented with symptoms of malaria at the healthcare facilities, 75 met the eligibility criteria, provided both written and verbal consent, and were subsequently enrolled in the study. However, only 52 patients successfully completed the study (Fig. 1).

### Sample size

It was estimated that the power of study posteriori based on the size of our sample applying the methods suggested by Ogungbenro and Aarons [29]. Based on the above the 52 subjects that completed the study provided adequate AL parent drug and metabolite data to demonstrate differential drug plasma levels.

### Malaria diagnosis and AL treatment

Participants were recruited from the Cape Coast Teaching Hospital, Ewim Polyclinic and Moree Health Post, all within the Cape Coast Metropolis, Central Region, Ghana. Verbal and written informed consent were obtained from participants after the study had been explained to them in both English and local languages (Fante and Twi). Patients were initially diagnosed with malaria RDT at the Outpatient Department (OPD) of the recruiting facilities, confirmed with microscopy by a microscopist and placed on any of the available brands of generic artemisinin-based combinations for three days. Follow up calls were made to participants for them to take their medications on time. AL 80 mg/480 mg was taken after meals 8–12 hourly for a 3-day regimen. Thin and thick-smear blood films were stained with Giemsa stain and analysed under a microscope for the presence of parasites. Blood samples were taken on days 1, 2, 3 and 7 for parasitaemia and blood metabolite analysis. Samples were taken not more than 2-h post dosing due to the short half life of artemether. Plasma samples were stored in − 80 °C until ready for analysis.

### Haematological analysis

Five millilitres of venous blood was taken into EDTA tubes for full blood count (FBC) analysis on a Sysmex-XN-350-15024 haematological analyzer.

### DNA extraction and genotyping

DNA was extracted from whole blood using the EZNA DNA extraction kit (Omega Bio-tek, Inc. Norcross, USA) according to the manufacturer’s instructions. The quality of DNA was established on a 1% agarose gel and nanodrop. Genotyping for CYP2B6*6, CYP2B6*18, CYP3A4*2, CYP3A4*17, CYP3A4*22, CYP3A5*2, CYP3A5*3, CYP3A5*6 and CYP3A5*7 was undertaken using Iplex GOLD SNP genotyping protocol on the Agena MassARRAY® system (Agena BioscienceTM, San Diego, CA, USA).

### Analysis of plasma concentrations of artemether, dihydroartemisinin (DHA), lumefantrine and desbutyl-lumefantrine (DBL)

#### Sample preparation

For the extraction of lumefantrine and desbutyl lumefantrine from plasma, a volume of 400 µL of acetonitrile containing lumefantrine-d9 at 15 ng/mL was added to 100 µL of plasma. The sample was vortex-mixed for 30 s and centrifuged at 15,000×*g* for 5 min at room temperature. A volume of 200 µL of the supernatant was transferred into a 96-well plate for analysis. For the extraction of artemether and its metabolite from plasma, a volume of 100 µL of plasma was added to a polypropylene microcentrifuge tube, together with 100 µL of 20 mM ammonium bicarbonate containing artemether-d3 and dihydro artemisinin-d3 at 100 ng/mL. Following the addition of 700 µL of ethyl acetate, the sample was vortex-mixed for 1 min and centrifuged at 16,000×*g* for 5 min at 4 °C. A volume of 650 µL of the top organic layer was transferred into a glass tube and dried under a gentle stream of nitrogen at 30 °C. The sample was reconstituted with 150 µL of methanol: 10 mM ammonium acetate (65:35; v:v) containing 0.1% acetic acid and vortex-mixed for 30 s prior to transfer into a 96-well plate for analysis.

#### Equipment

Analysis for artemether, DHA, lumefantrine and DBL was undertaken at the Division of Clinical Pharmacology, University of Stellenbosch, Cape Town, South Africa. Liquid chromatography–mass spectrometry (LC–MS/MS) analysis was conducted on a SHIMADZU 8040 triple quadrupole-mass spectrometer (SHIMADZU, Kyoto, Japan) connected to a SHIMADZU Prominence LC system. The system consisted of a LC-20ADXR solvent delivery system, Nexera XR SIL-20AXR autosampler and CTO-20A column oven. The analytes were chromatographically resolved on an Agilent Poroshell 120 EC-C18 (3.0 × 100 mm, 2.7 µm) column. Data acquisition and processing was performed using LabSolutions Version 5.109 software (Shimadzu Corporation, Kyoto, Japan).

#### Analysis

Artemether and dihydroartemisinin were quantified as described by Wiesner et al. [30], with modifications. Lumefantrine and desbutyl lumefantrine were quantified as described by Govender et al*.* [31], with modifications. The liquid chromatography-tandem mass spectrometry (LC–MS/MS) methods were validated according to Food and Drug Administration (FDA) [32] and European Medical Agency (EMA) [33] guidelines prior to sample analysis.

### Statistical analysis

Descriptive statistics of participants including generic brands, parasitiemia, medians and means full blood count parameters was performed. Continuous variables were expressed as mean ± standard deviation or median (inter-quartile range), with categorical variables being expressed as absolute values and or frequencies. Kruskall Wallis or Dunn’s test was used to test for significance among various groups. Linkage disequilibrium, haplotype genotype and allele frequencies were calculated using a web based tool LDlink [34] and Shesis Plus [35]. A p value of p < 0.05 was considered statistically significant. Statistical analysis was performed using STATA v18 ((StataCorp, College Station, Texas, USA) and Graphpad v9 (Prisma, San Diego, California) statistical software packages.

## Results

### Clinico-demographic characteristics

Table 1 shows the clinicodemographic data for the study participants. There were more females (61.54%) than males (38.46%). Mean age was 34.83 ± 18.32 years for both males and females. Lumetrust (42.31%) and Lumether (28.85%) were the most administered generic artemisinin-based combinations brands to participants. Full blood count (FBC) analysis showed a mean value for Hb (11.37 ± 1.94 g/dL), HCT (36.43 ± 8.06), PLT (166.67 ± 88.20) × 10^3^/µL) and WBC (4.60 ± 2.76) × 10^9^/L). On day 1 of recruitment, the median parasite density for participants was 2666.67/µL of blood, while on day 2, the parasite count was 1529.89/µL; by day3, parasite density was 0.

**Table 1:** General clinicodemographic of patients on ACT

	Total number of participants, N = 52
Gender	
Male	20 (38.46)
Female	32 (61.54)
Mean age in years ± SD (range)	34.83 ± 18. 32 (15–78)
Median BMI in kg/m^2^ (IQR)	22.10 (19.36–26.85)
Mean Hb in g/dL ± SD (range)	11.37 ± 1.94 (7.1–15.8)
Mean HCT (%) ± SD (range)	36.43 ± 8.06 (9.8–55.90)
Mean PLT (10^3^/µL) ± SD (range)	166.67 ± 88.20 (35–369)
Mean WBC (10^9^/L) ± SD (range)	4.60 ± 2.76 (0.86–16.61)
ACT BRANDS n (%)	
Artetab	8 (15.38)
Lumether	15 (28.85)
Lumetrust	22 (42.31)
Luzatil	4 (7.69)
Shal'Artem	3 (5.77)
Parasite density (parasite/μL of whole blood), median, IQR	
Day 1	2666.67 (397.62–10,687.57)
Day 2	1529.89 (36.86–4804.24)
Day 3	0
Day 7	0

**Fig. 2:**
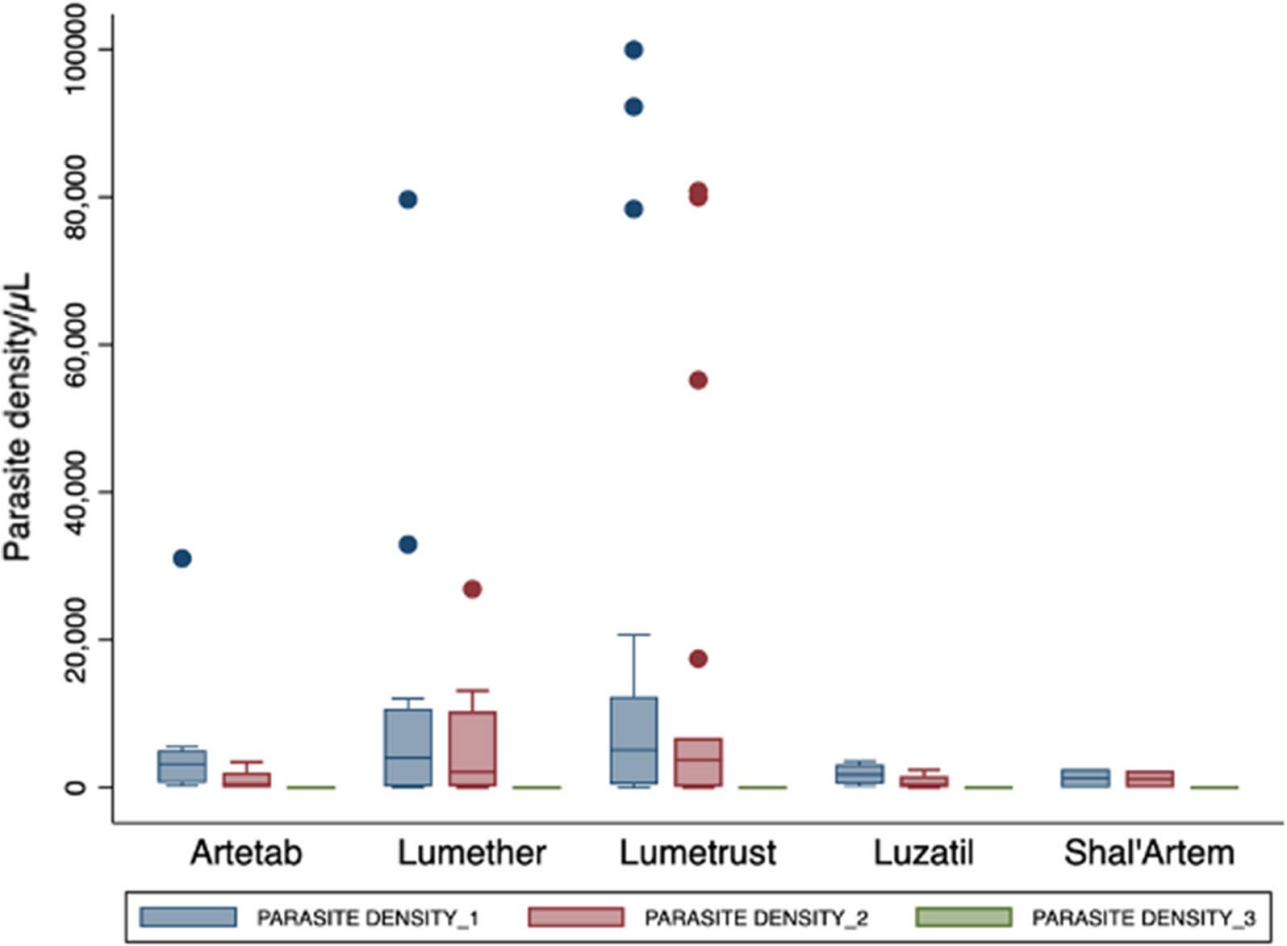
Parasite density per generic ACT administered. Day 1 to 3 parasite density as quantified by microscopy per administered ACT

Figure 2 shows the parasite density in relation to the various artemisinin-based combinations that participants took, and it shows that despite the differences in observed parasite density, the trend of 0 parasite density on day 3 and 7 was seen for all treatments.

### Plasma artemether–lumefantrine concentration distribution among the generic brands

Plasma concentrations of artemether, dihydroartemisinin, lumefantrine and desbutyl lumefantrine influences parasitaemia response and treatment outcome in malaria. Parasite resistance can set in at significantly low levels, while higher plasma concentrations may also likely be associated with adverse drug events. This study successfully gathered comprehensive drug and metabolite plasma concentrations from all 52 samples. However, it is essential to note that for three of these patients, the drug concentrations of some metabolites were below the detectable limit. High plasma concentrations of lumefantrine and desbutyl lumefantrine were observed across all brands of generic AL. However, the high plasma concentration for lumefantrine and desbutyl lumefantrine were observed in participants who were dosed with Artetab, 4123.75 (3056.34–4943.79) ng/mL and 35.87 (15.37–71.15) ng/mL, respectively. The day 7 plasma lumefantrine concentration for all brands of generic AL were above 200 ng/mL, which could account for the parasitaemia levels observed (Table 2).

**Table 2:** Generic brands of ACT parent drug and metabolite concentrations

ACT and metabolites (ng/mL)	Artetab	Lumether	Lumetrust	Luzatil	Shal'Artem	P value
	Median (IQR)					
Artemether	23.90 (17.70–30.95)	22.37 (9.89–49.40)	12.20 (6.09–17.10)	8.75 (N/A)	16.03 (N/A)	0.4832
Dihydro artemisinin (DHA) (2-h postdose)	35.87 (15.37–71.15)	15.84 (9.62–32.31)	32.98 (12.02–64.94)	N/A	31.47 (N/A)	0.4161
Lumefantrine concentration—day 3	4123.75 (3056.34–4943.79)^a,b,c^	3024.97 (2118.00–3926.77)	2538.58 (1727.43–3742.91)	1940.07 (N/A)	3235.86 (500.00–5971.7)	0.0202*
Desbutyl lumefantrine—day 3	44.10 (24.76–77.42)^d,e^	24.07 (14.19–40.28)	23.58 (14.11–43.28)^f^	19.44 (14.28–24.6)	27.79 (14.28–54.90)	0.0664
Lumefantrine concentration—day 7	678.83 (456.44–1171.07)	583.0 (454.01–1585.06)	550.77 (351.14–1151.70)	692.03 (N/A)	606.16 (250.00–962.33)	0.3323
Desbutyl lumefantrine (DBL)—day 7	39.32 (24.29–60.03)^g,h^	32.64 (14.60–51.82)	17.05 (11.30–24.24)	39.4 (N/A)	36.37 (34.00–38.73)	0.0715

N/A-some values were below lowest limit of quantification (LLOQ), Statistically significant comparisons among the generic drugs—^a,b,c^comparison between Artetab, Lumether, lumetrust, Shal’Artem; ^d,e^comparison between Artetab, Lumether, Lumetrust; ^f^Lumether, Shal’Artem; ^g,h^comparison between Artetab, Lumetrust

### Correlation between CYP2B6 and CYP3A5 genotypes with plasma concentrations of artemether, DHA, lumefantrine and desbutyl lumefantrine metabolites

Table 3 provides a detailed analyses of the *CYP2B6* and *CYP3A5* genotypes, highlighting the variations in plasma DHA concentrations across different genotypes. On day 3, a significant difference in plasma DHA concentrations was noted among the various CYP2B6*6 genotypes (p = 0.0031*). Additionally, on day 7, there was a notable difference in plasma DBL concentrations (p = 0.0019*) between CYP3A5*6 carriers. Desbutyl lumefantrine is the potent metabolite of lumefantrine with significant anti-malarial activity. The median day 3 plasma lumefantrine and desbutyl lumefantrine concentrations were 2979.25 (1869.30–4123.75) ng/mL and concentration of desbutyl lumefantrine 27.79 (14.28–44.76) ng/mL, respectively while that of day 7 was 578.18 (367.62–1057.01) ng/mL and 24.24 (14.60–39.32) ng/mL, respectively.

**Table 3:** Plasma artemether–lumefantrine and metabolite concentrations stratified by genotypes

Genotype	n (%)	Median (IQR)											
		ART (ng/mL)	p-value	DHA (ng/mL)	p-value	LUM-DAY 3 (ng/mL)	p-value	DBL-DAY 3 (ng/mL)	p-value	LUM-DAY 7 (ng/mL)	p-value	DBL-DAY 7 (ng/mL)	p-value
CYP2B6 rs28399499 (*18)													
T/T	47	48.50 (14.46–99.14)	0.1627	29.68 (11.45–52.80)	0.7937	3004.72 (1823.08- 4502.25)	0.3699	26.28 (14.28–44.43)	0.2887	573.36 (381.45- 1151.70)	0.7231	22.90 (14.60–39.32)	0.9587
C/T	5	8.98 (6.03–71.61)		51.08 (4.75–97.40)		2029.04 (1929.00–2573.97)		41.8 (24.60–55.75)		613.00 (339.52–693.03)		29.09 (16.04–36.58)	
CYP2B6 rs3745274 (*6)													
T/T	15	40.47 (9.89–111.40)	0.8509	29.43 (16.30–54.20)	0.0031*	2989.45 (1823.08–4893.64)	0.9610	17.64 (15.70–61.48))	0.7244	469.34 (353.78–689.49)	0.3315	14.23 (11.30–42.00)	0.5937
G/T	23	32.86 (13.68–86.07)		13.32 (8.12–32.30)		3029.94 (1903.77–3926.77)		26.77 (14.28–47.12)		693.45 (500.00–1262.36)		23.57 (18.28–33.75)	
G/G	14	69.24 (10.71–113.95)		90.90 (44.35–111.33)		2488.28 (1929.00–4335.37)		34.20 (24.60–41.80)		613.00 (495.72–918.00)		33.43 (16.54–39.40)	
CYP3A5 rs776746 (*3)													
T/T	38	42.39 (12.28–102.79)	0.7335	29.68 (9.67–66.57)	0.8102	2926.18 (1869.30–4123.74)	0.5594	27.79 (14.28–50.14))	0.3506	583.00 (353.78–1151.70)	0.7214	24.29 (15.19–39.40)	0.1776
T/C	14	55.77 (9.21–92.80)		29.26 (16.30–34.37)		3648.10 (1970.54–4238.41)		22.59 (13.04–33.14)		559.91 (381.45–918.00)		15.46 (11.97–25.46)	
CYP3A5 rs41303343 (*7)													
−/−	37	37.37 (9.89–111.40)	0.8287	16.30 (5.90–49.68)	0.6569	3020.00 (1869.30–4547.00)	0.2991	29.48 (14.28–50.14)	0.5114	583.00 (342.81–1220.00)	0.7613	24.69 (13.93–40.66)	0.5094
−/T	15	59.17 (16.52–93.91)		30.25 (8.36–44.35)		2664.17 (1940.07–3056.34)		24.68 (16.91–32.33)		573.36 (550.39–692.03)		18.99 (16.54–33.42)	
CYP3A5 rs10264272 (*6)													
C/C	39	48.50 (10.9–99.14)	0.6084	19.75 (5.89 -49.68)	0.9800	2989.45 (1903.88–3926.77)	0.8284	17.23 (14.28–36.79)	0.0019*	573.36 (454.01–1220.00)	0.9353	19.44 (13.27–33.75)	0.3424
C/T	13	28.34 (10.24–80.96)		18.78 (8.08–49.83)		2795.56 (1592.93–4893.64)		47.45 (31.60–77.42)		689.49 (342.81–962.33)		35.43 (17.57–39.32)	

^*^Significant

### Correlation between CYP2B6 and CYP3A5 expressor status with plasma concentrations of artemether, DHA, lumefantrine and desbutyl lumefantrine metabolites

**Fig. 3:**
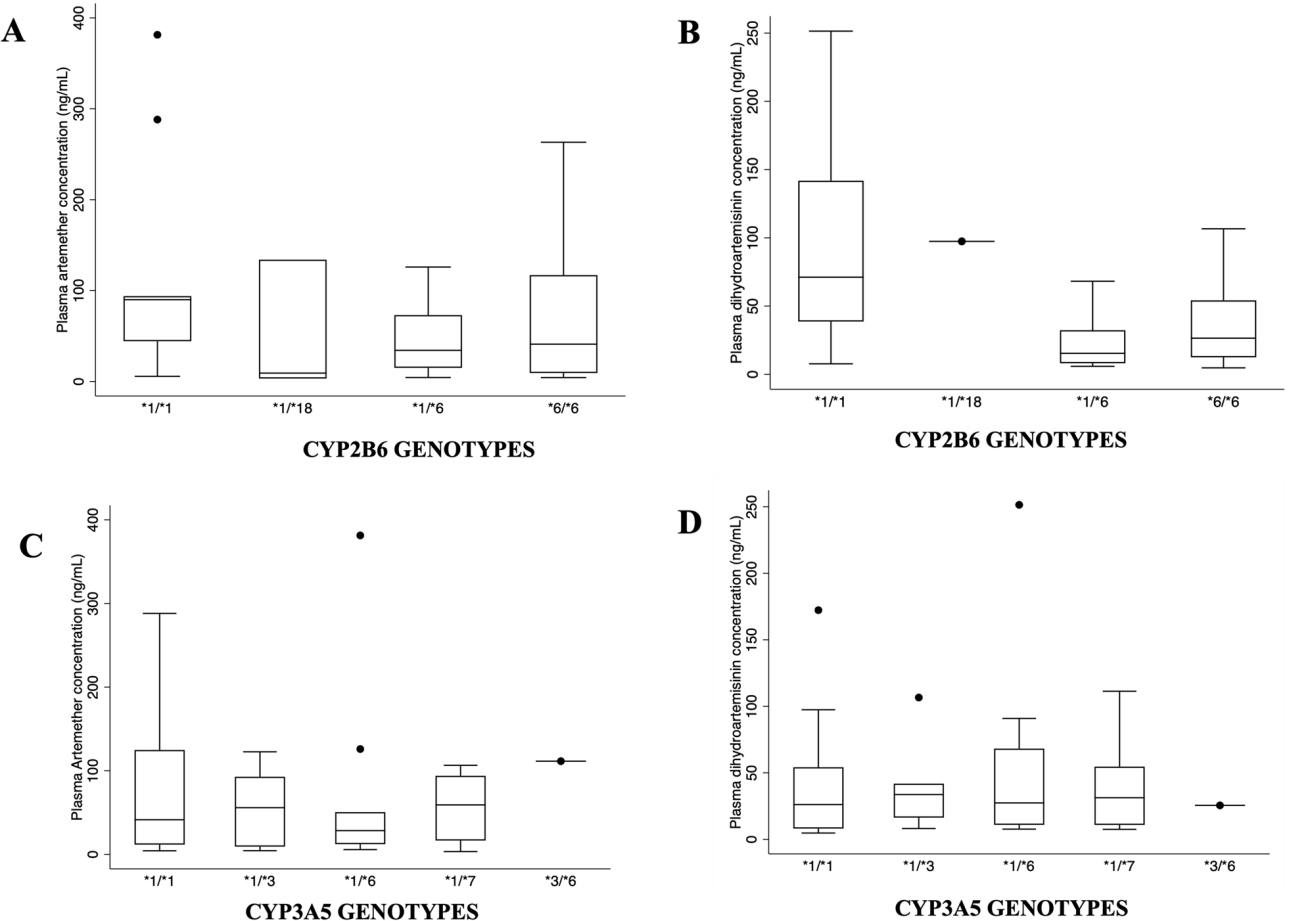
Plasma drug concentration of artemether and dihydroartemisinin and CYP2B6 and CYP3A5 expressors. **A** Observed significance between *1/*6 vs *1/*1 (*p* = *0.039*). **B** observed significance between *1/*6 vs *1/*1 (*p* = *0.000*), *6/*6 + *18/*18 vs *1/*1 (*p* = *0.012*). **C** There were no observed significant differences. **D** There were no observed significant differences

The study observed considerable inter-individual variability in the plasma concentrations of artemether, dihydroartemisinin, lumefantrine and desbutyl lumefantrine although most variations were not statistically significant. This lack of significance could be attributed to the sample sizes used in the analyses. Median plasma concentrations of artemether and its metabolite, dihydroartemisinin, ranged from 8.98–69.24 ng/mL and 16.30–90.90 ng/mL, respectively, while median plasma concentrations of lumefantrine and desbutyl lumefantrine ranged from 2664.17–3029.94 ng/mL and 17.23–47.45 ng/mL respectively for day 3 and 469.43–689.49 ng/mL and 14.23–35.43 ng/mL, respectively, on day 7. There were 4 samples where the metabolite concentration for LUM and DBL for day 7 were below the lower limit of quantification. The analysis combined non-expressor CYP2B6*6/6 and *18/*18 due to their numbers and functional effects. Figure 3 illustrates plasma artemether and DHA concentrations in relation to CYP2B6 and CYP3A5 expressors. There were significant differences in plasma artemether drug concentrations between CYP2B6*1/*6 and *1/*1 carriers (*p* = *0.039*) with another significant difference observed between plasma DHA metabolite concentrations between CYP2B6*1/*6 versus CYP2B6*1/*1 (*p* = *0.000*) and combined CYP2B6*6/*6 + *18/*18 versus CYP2B6*1/*1 (*p* = *0.012*).

**Fig. 4:**
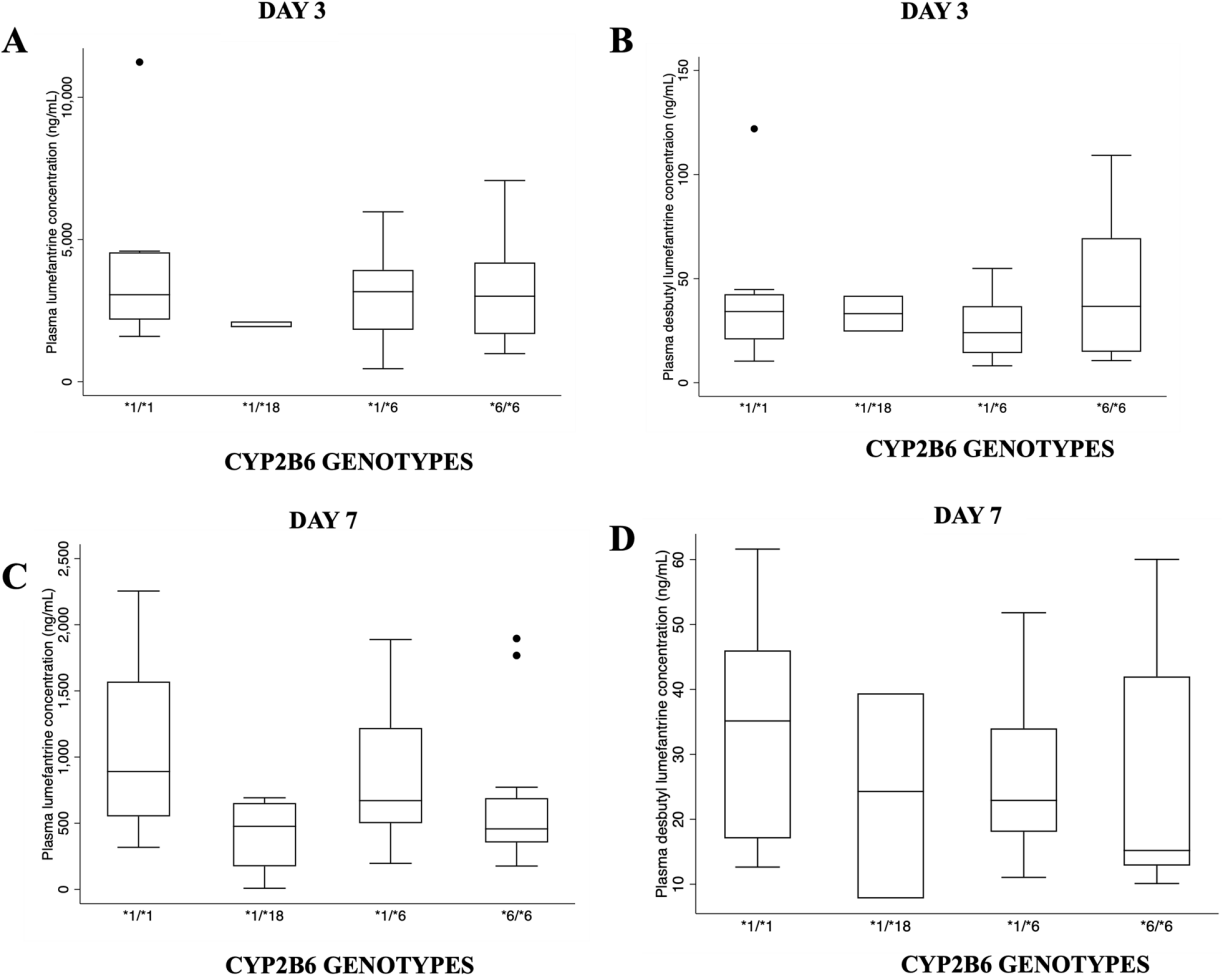
Plasma drug concentration of lumefantrine and desbutyl lumefantrine and CYP2B6 expressors on day 3 and 7. **A** There were no observed significant differences. **B** There were no observed significant differences. **C** There were no observed significant differences. **D** There were no observed significant differences

Plasma lumefantrine and DBL in relation to *CYP2B6* is shown in Fig. 4. No significant difference was observed between normal expressor (*1/*1) and reduced expressors. However, it was observed that the median plasma concentration for normal expressor *1/*1 was relatively higher for day 7 plasma lumefantrine and desbutyl lumefantrine in comparison to reduced expressors.

**Fig. 5:**
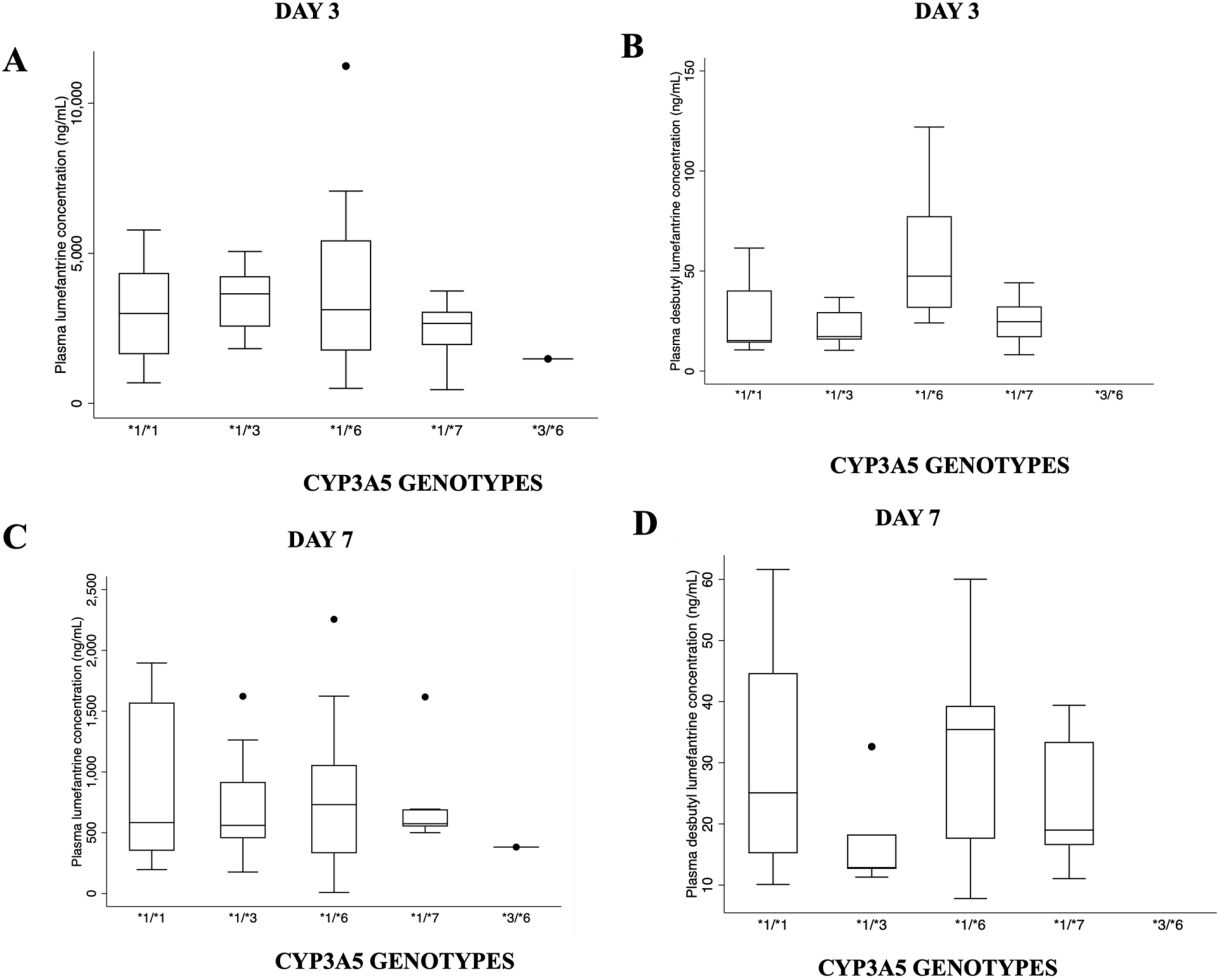
Plasma drug concentration of lumefantrine and desbutyl lumefantrine and CYP3A5 expressors on day 3 and 7. **A** There were no observed significant differences. **B** Observed significance between *1/*6 vs *1/*1 (p = 0.002), *1/*6 vs *1/*3 (p = 0.006), *1/*7 vs *1/*6 (p = 0.008). **C** There were no observed significant differences. **D** Observed significance between 1A/*6 vs 1A/*3 (p = 0.026)

Figure 5 shows the plasma lumefantrine and DBL for *CYP3A5* expressor status. There were significant differences in plasma DBL concentrations on day 3 between *1/*1 versus *1/*6 (*p* = *0.002*), *1/*3 versus *1/*6 (*p* = *0.006*) and *1/*7 versus *1/*6 (*p* = *0.008*). There was an observed significance on day 7 desbutyl plasma concentrations among *1/*6 and *1/*3 (*p* = *0.026*) expressors.

**Fig. 6:**
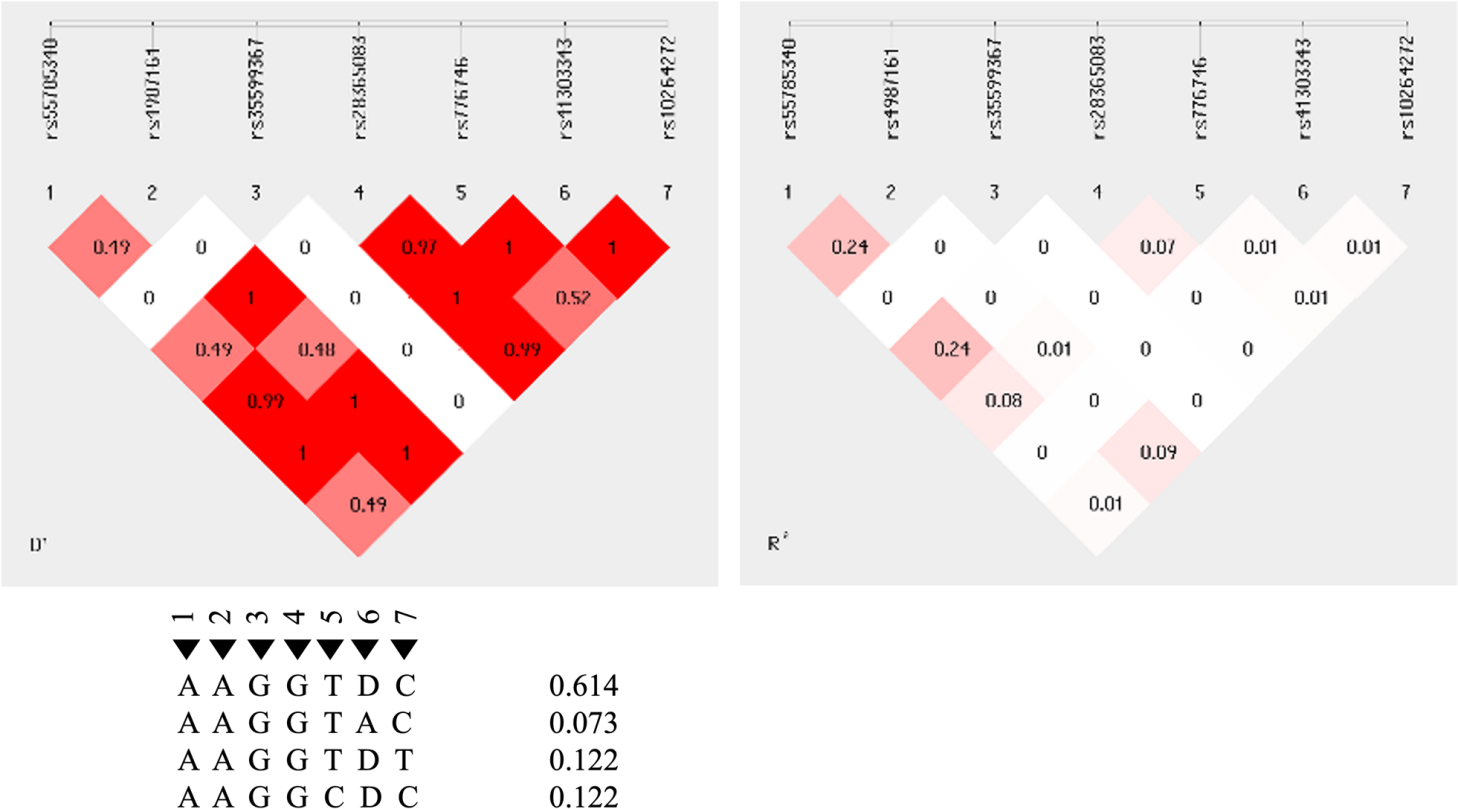
Linkage disequilibrium (LD) plot of CYP3A4 (g.15713 T > C), CYP3A4 (T15615C), CYP3A4 (g.15389 C > T),CYP3A5 (C27289A), CYP3A5 (A6986G), CYP3A5 (g.14690 G > A), CYP3A5 (g.27131-27132insT) and observed D’ and R’ values. The pair-wise LD association between two SNPs and the corresponding D’ and R’ values. The colour gradient from red to white reveals higher to lower LD (D’ 1–0; R’ 1–0)

The linkage disequilibrium (LD) observed among *CYP3A4* and *CYP3A5* variants were from low to high (Fig. 6). CYP3A4*22 and CYP3A5*6 were in high LD while all the *CYP3A5* variant alleles (*3, *6,*7) occur in high LD which is similar to observations in a study undertaken in pregnant women in Tanzania [36].

## Discussion

The therapeutic efficacy of AL is largely dependent on the systemic bioavailability of active metabolites [25, 26]. Clinical dosing guidelines for AL are based on body weights with a fixed artemether to lumefantrine ratio of 1:6. However, plasma drug and metabolite concentrations and their effect on parasitaemia have not been thoroughly studied in most exposed populations [37]. In Ghana, there is no data on the plasma drug and metabolite concentration for patients who are prescribed AL and how it influences parasitaemia. This study is the first to evaluate cytochrome P450 variation on plasma drug and metabolite concentration of generic AL and its effects on parasitaemia in Ghanaians. This study, therefore, report the first data on the effects of pharmacogenetic variations on plasma concentrations of artemether and lumefantrine in patients who presented with uncomplicated malaria at health facilities. The most notable finding is that although pharmacogenetic variations influence plasma concentrations of AL and its metabolites, parasitaemia significantly reduces with strict adherence to AL.

Coartem® (Novartis Pharma AG, Basel Switzerland) is the pioneer innovator of artemether lumefantrine approved by the WHO in 2006, which has been a very effective anti-malarial drug [38]. However Coartem® is sold at an average price of US$ 4.5–6, which poses financial barriers affecting its availability and affordability negatively [39]. Generic ALs have been prequalified to be used in the treatment of uncomplicated malaria in several African countries. In this current study, the pharmacogenetics of generic AL plasma drug and metabolite concentration and treatment outcome in uncomplicated malaria is investigated in patients who are on generic AL, which is mostly prescribed at health facilities in Ghana. Functional variants of cytochrome P450 enzymes which are responsible for AL metabolism, namely *CYP2B6*, *CYP3A4* and *CYP3A5,* were evaluated. Major findings from the study are that *CYP3A5* genotypes influences plasma AL metabolism and thus plasma concentrations whereas CYP3A5 *3 and *6 is associated with elevated levels of AL and metabolites. While several studies have explored effect of pharmacogenetics on AL [20, 36, 40] treatment in different populations, this study is unique in its evaluation of cytochrome P450 variation on plasma metabolite concentrations of generic AL and its effects on parasitaemia in Ghanaians. Understanding the role of pharmacogenetics in drug metabolism is crucial, especially in discerning how variations in key drug-metabolizing enzymes involved in AL metabolism might influence drug disposition and parasite clearance. This knowledge is vital for optimizing malaria treatment and ensuring effective patient care.

Artemether continues to be one of the most administered anti-malarial medications globally over the past 20 years, having been adopted by the majority of National Malaria Control Programmes in Africa [41]. Artemether metabolism is primarily mediated by *CYP2B6* [42, 43], with secondary contribution by *CYP3A4/5*. Our study showed there was substantial interindividual variability among the participants. As part of the analysis and looking at the genotype frequencies obtained for CYP2B6*6/*6 and CYP2B6*18/*18, they were combined in our analysis since they both have similar functional effects. Day 3 and 7 plasma metabolite concentrations for variations in *CYP2B6* showed reduced artemether and DHA metabolite concentrations for the combined CYP2B6 *6/*6 + CYP2B6 *18/*18 genotypes (Fig. 2). The effect on metabolic ratio is that these genotypes *6/*6 + *18/*18 have higher metabolic ratios than *1/*1 carriers. The observation in our study is similar to a study which found a significant increase in the metabolic ratio of artemether-to-dihydroartemisinin of CYP2B6*6/*6 volunteers over their *1/*1 counterparts [40]. There is less pharmacokinetic data on dihydroartemisinin (DHA), which is considered a very potent metabolite of artemether, and in this study, we quantified plasma DHA and observed a median concentration range of 11.45–90.90 ng/mL. A previous study has shown declining artemether and DHA concentrations over time, with these compounds becoming undetectable at < or = 18 h [44]. In contrast, another 12-h post-dose analysis study found the median concentration of DHA ranging from 54–158 ng/mL for patients with sensitive responses [37], which was important for recrudescence although it has a very short half-life.

Plasma lumefantrine concentration for CYP3A5 *1/*1 was lower than other genotypes such as *1/*3, *1/*6, *1/*7 and *3/*6. There have been limited studies undertaken on *CYP3A4*-*CYP3A5* genotypes and their effects on AL, however, the role of *CYP3A4* and *CYP3A5* in artemether and DHA metabolism is highly elucidated [45, 46]. One of the determinants of plasma artemether and LUM concentrations is the *CYP3A* pharmacogenetic status of an individual. CYP3A4*1B has been shown to be significantly associated with especially day 7 plasma LUM concentrations affecting treatment outcome [36]. All the CYP3A4 genotypes studied, including *2, *17 and *22, were monomorphic and, therefore, excluded from the analysis. A previous study in Ghana also genotyped CYP3A4*1B and found monomorphism [47], which could mean variation in this enzyme in Ghanaian population could be limited. A future study would incorporate other *CYP3A4* variations for consideration.

Lumefantrine is the long acting component of AL and is metabolized to a more active component, desbutyl lumefantrine, which CYP3A4/5 principally metabolizes with an extensive anti-malarial activity [17]. Lumefantrine is absorbed and cleared more slowly with a half-life of 3–4 days and its concentration accumulates with successive doses, therefore preventing recrudescence [18]. Evaluation of the data showed monomorphism in the three *CYP3A4* and CYP3A5*2 variants, so the effect of CYP3A5 *3, *6 and *7 variants on the plasma metabolite levels of lumefantrine from dosed generic AL were rather analysed. However, with the knowledge that CYP2B6 is capable of metabolizing 25%-30% of currently known clinical drug substrates of CYP3A4/5 [48, 49], analysis of the effects of *CYP2B6* variation on lumefantrine metabolism as part of this study was undertaken. There were observed variations in *CYP2B6* genotype effects on lumefantrine and desbutyl lumefantrine concentrations (Fig. 3). The median plasma lumefantrine concentration on day 3 was 2773.74 (1401, 3811.20) ng/mL and there was significance in day 3 median plasma desbutyl lumefantrine concentration between *1/*1 carriers and *1/*6 carriers.

The influence of *CYP3A5* haplotypes on lumefantrine plasma concentrations is shown in Fig. 4. There were variable effects on the lumefantrine plasma metabolite levels where there were significant differences between *1/*1 and *1/*6 carriers. CYP3A5 *1/*1 carriers had reduced plasma lumefantrine and DBL concentrations on day 3 compared to other variations. Similar patterns of reduced lumefantrine and DBL concentrations were observed on day 7. A recent study in Tanzania reported that CYP3A5 *1/*1 genotypes are significantly associated with low plasma lumefantrine concentrations [50] while another study also reported defective alleles, such as CYP3A5*3 variant, are associated with high plasma lumefantrine concentrations [51]. A previous study reported that CYP3A5 *1/*1 genotype had a significantly higher risk of having plasma lumefantrine concentration of < 600 ng/mL [36] and it is observed in this study that *1/*1 carriers had a median plasma lumefantrine concentration of < 600 ng/mL (Fig. 5) on day 7 which may be associated with risk of recurrent parasitaemia.

Several studies have previously reported that day 7 plasma lumefantrine concentrations significantly influence recrudescence. A systematic review using individual patient data concluded that day 7 plasma concentrations of ≥ 200 ng/mL is associated with greater cure rates [52] while other studies reported that day 7 lumefantrine concentrations < 600 ng/mL is associated with treatment failure [36]. This study observed that the median day 7 plasma LUM concentrations for all generic lumefantrine medications prescribed to our patients was 578.17 (367.62–1057.01) ng/mL. Irrespective of the medication that was prescribed, there were observed significant decline in parasite density from day 2 through day 7 (Fig. 2). This implies that these generic artemisinin-based combinations prescribed could have enough anti-malarial activity to clear the malaria parasite when there is adherence to the prescribed dosage regimen.

Desbutyl-lumefantrine, the metabolite of lumefantrine, has shown greater anti-malarial potency and synergy with lumefantrine and artemisinin. A study showed DBL has effective activity against field isolates and laboratory strains of *P. falciparum* at a concentration of 15.5 ng/mL (0.6–58.20) [19]. In this study, the median plasma DBL concentration observed f ranged from 17.23–47.45 ng/mL on day 3 and 14.23–35.43 ng/mL on day 7 (Table 3). From these observations, there should be a certain plasma lumefantrine concentration to reach sufficient DBL concentrations to influence treatment outcomes.

Results therefore show that pharmacogenetic variations in *CYP2B6* and *CYP3A5* influence plasma disposition of artemether and lumefantrine and likely affect malaria treatment outcome if medication is not adhered to. Knowing the significant influence of CYP3A4 and CYP3A5 on AL metabolism, the interaction between these two CYP3A genotypes may largely determine eventual plasma exposure and parasite clearance *vis-a-vis* treatment outcome. Given that CYP3A4 and CYP3A5 are found within the same gene block, and there could be several major haplotype combinations in the CYP3A4-CY3A5 block [53], observed effects in terms of plasma concentrations that might be attributed to *CYP3A4* allele might actually be due to the influence of a *CYP3A5* allele in LD. There was linkage disequilibrium (LD) among *CYP3A4* and *CYP3A5* alleles, and following some of the monomorphisms observed in CYP3A4 in this study, it can be hypothesized that the association between CYP3A4 and CYP3A5 play a critical role in plasma AL concentration.

This study faced limitations, particularly in terms of sample size, which, while adequate for assessing anti-malaria drug efficacy, could have been larger for more comprehensive results. Additionally, different brands of ACT were used and there was an uneven distribution of patients across different generic AL brands due to the study's reliance on health facilities for recruitment, leaving the researchers with no control over the specific ACT brands dispensed. Despite these challenges, the study successfully underscores the significance of pharmacogenetic variations in influencing plasma AL concentrations demonstrating the effectiveness of the generic AL brands used in this study when the dosage regimen is properly followed.

## Conclusion

The study findings show that variations in *CYP2B6* and *CYP3A5* enzymes may lead to higher lumefantrine exposure across selected generic artemether–lumefantrine brands dispensed. It is, therefore, important to investigate the impact of pharmacogenetic variations, including *CYP3A* haplotypes, on artemether–lumefantrine in a larger cohort in addition to other parameters such as pregnancy, concomitant drug use and comorbidities/infections in the Ghanaian population.

## Data Availability

Raw data from this study is available upon reasonable request from the corresponding author.

## Abbreviations

ACT: Artemether–lumefantrine combination therapy
RDT: Rapid detection test
AL: Artemether–lumefantrine
DHA: Dihydroartemisinin
DBL: Desbutyl-lumefantrine
AMFm: Malaria Initiative
LMIC: Low- and middle-income countries
OPD: Outpatient Department
LC–MS/MS: Liquid chromatography-tandem mass spectrometry
FDA: Food and Drug Administration
EMA: European Medical Agency
LLOQ: Lower limit of quantification
